# Levodopa-accelerated frailty: a hypothesis for a cumulative iatrogenic burden in Parkinson’s disease

**DOI:** 10.3389/fneur.2026.1798514

**Published:** 2026-04-15

**Authors:** Claus Skaaning

**Affiliations:** Independent Researcher, Aalborg, Denmark

**Keywords:** frailty, iatrogenic, levodopa, non-motor symptoms, Parkinson’s disease

## Abstract

Levodopa remains the cornerstone of symptomatic treatment in Parkinson’s disease, providing substantial motor benefit and improved quality of life. While epidemiological studies have suggested improved survival in the modern levodopa era compared with the pre-levodopa era, these observations derive primarily from observational cohorts rather than randomized trials. Yet many patients experience severe functional decline after 5–10 years, a paradox that challenges the reputation of our most potent therapy. Similar divergences between early symptomatic benefit and adverse long-term outcomes have been observed in other areas of medicine. For example, positive inotropic agents such as milrinone improved short-term exercise tolerance in heart failure but were later associated with increased mortality in long-term trials. Antiarrhythmic agents encainide and flecainide effectively suppressed ventricular arrhythmias yet increased mortality in the Cardiac Arrhythmia Suppression Trial (CAST). In diabetes, intensive glucose lowering strategies improved metabolic control but increased cardiovascular mortality in the ACCORD trial, and earlier studies of tolbutamide in the University Group Diabetes Program (UGDP) also suggested excess cardiovascular deaths despite improved glycemic control. These examples illustrate how therapies with clear short-term physiological benefits may reveal unanticipated risks when evaluated over longer time horizons. Similarly, the long-term use of levodopa may introduce a cumulative burden of complications that may independently accelerate decline. We propose the conceptual framework of *levodopa-accelerated frailty* as a testable hypothesis to explore this possibility. This framework synthesizes evidence across seven pathways that may interact or overlap in contributing to frailty: dyskinesia, psychosis, orthostatic hypotension, weight loss, impulse control disorders, sleep disturbances, and elevated homocysteine. Each complication has been associated with increased risks of dementia, hospitalization, falls, or mortality in observational studies, with reported hazard ratios generally ranging from approximately 1.5 to over 6. Together, they form a synergistic web of decline that may transform a highly effective symptomatic therapy into a contributor to late-stage vulnerability. This hypothesis reframes a potential therapeutic paradox: a treatment that improves early symptomatic outcomes may also interact with mechanisms that contribute to frailty later in the disease course. The clinical implication is not levodopa phobia, but a healthspan-preservation strategy focused on minimizing cumulative iatrogenic burden. This involves adhering to the lowest effective dose, continuous reassessment, and integration with exercise, nutrition, and adjunctive therapies. Recognizing this potential pattern of levodopa-accelerated frailty may help reconcile the discrepancy between early symptomatic success and the later emergence of vulnerability. We emphasize that this model is a hypothesis and call for long-term, prospective studies to test whether cumulative levodopa exposure contributes to frailty and reduced healthspan.

## Graphical abstract

**Figure:**
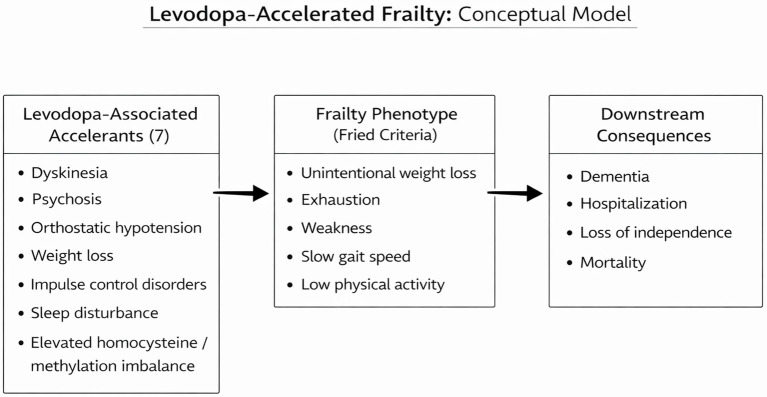
Conceptual model of levodopa-accelerated frailty linking seven levodopa-associated accelerants to established frailty phenotype criteria (Fried phenotype) and downstream outcomes. The framework is hypothesis-generating and illustrates how treatment-associated complications may interact with frailty mechanisms to increase vulnerability to dementia, hospitalization, loss of independence, and mortality.

## 1 Introduction: the levodopa paradox and the challenge of confounding by indication

Levodopa remains the cornerstone of symptomatic treatment in Parkinson’s disease and is widely regarded as the most effective therapy for motor symptoms, as reflected in clinical guidelines and randomized trials demonstrating substantial improvements in motor function and quality of life (1, 2).

Yet many patients experience severe functional decline after 5–10 years, a paradox that challenges the reputation of our most potent therapy (1, 3). This demands a critical re-evaluation of levodopa’s total long-term impact on disease trajectory. Similar divergences between early symptomatic benefit and adverse long-term outcomes have been observed in other areas of medicine. Milrinone improved short-term exercise tolerance in heart failure but increased long-term mortality, encainide and flecainide suppressed ventricular arrhythmias yet increased mortality in the Cardiac Arrhythmia Suppression Trial (CAST), and in diabetes both ACCORD and the University Group Diabetes Program (UGDP) showed that improved metabolic control did not necessarily translate into better long-term outcomes (37–40). Here we present a conceptual and testable hypothesis, that cumulative, long-term levodopa exposure may indirectly accelerate frailty through interconnected non-motor complications. The conceptual structure of the proposed hypothesis is summarized in the graphical abstract.

The landscape of PD is now understood to extend far beyond its motor manifestations (4). A complex spectrum of non-motor symptoms (NMS), including psychosis, autonomic dysfunction, sleep disorders, and cognitive decline, are integral to the disease and are often more disabling than the motor deficits themselves (4, 5). One commonly proposed explanation is confounding by indication (6): patients with aggressive PD subtypes need higher doses earlier, making levodopa a marker of rapid progression rather than its cause.

While confounding by indication is a possible contributing factor, this paper proposes that the indirect iatrogenic burden of levodopa’s known complications, distinct from the thoroughly disproven hypothesis of direct neurotoxicity, contributes independently to long-term decline. This paper fully accepts the pivotal findings of the LEAP and ELLDOPA studies that levodopa does not alter the underlying rate of neurodegeneration. The hypothesis presented here is not about neurotoxicity, but about the cumulative harm from secondary complications that are mechanistically linked to long-term, pulsatile dopaminergic therapy.

This viewpoint seeks to elucidate the mechanisms of that contribution. Cross-sectional analysis by Peball et al. (2019) found that a higher total daily levodopa equivalent dose (LEDD) was independently associated with frailty even after adjusting for disease duration and severity as measured by the Hoehn and Yahr stage (7). This finding is reinforced by a systematic review from Tan et al. (2020), which also identified an association between total LEDD and frailty in PD across multiple studies (8). This hypothesis builds on the confounding model, proposing that long-term pulsatile dopaminergic therapy, of which levodopa remains the cornerstone, may indirectly accelerate decline via a synergistic cascade of motor and non-motor complications (9). Once established, these are independent drivers of morbidity and mortality. Their combined effect may be multiplicative, producing a state defined as “levodopa-accelerated frailty.”

This framework highlights the potential need for future studies examining optimal dopaminergic treatment strategies, particularly in patients with young-onset Parkinson’s disease who may experience decades of cumulative exposure.

## 2 Pathways of iatrogenic burden: the accelerants

This hypothesis rests on a two-step chain: (1) long-term, pulsatile levodopa therapy is associated with specific clinical complications, and (2) these complications are independently associated with accelerated disease progression and mortality. Far from being rare occurrences, these pathologies are highly prevalent in patients with long-standing PD undergoing chronic levodopa therapy. The following sections describe seven clinical and biological factors that may function as potential ‘accelerants’ within the proposed framework, presenting corrected and verifiable quantitative data on their profound prognostic impact, while acknowledging that causality remains unproven and requires validation through prospective study.

These factors should be viewed as illustrative rather than exhaustive, and additional mechanisms may contribute to frailty trajectories in Parkinson’s disease.

### 2.1 Levodopa-induced dyskinesia (LID)

Levodopa-induced dyskinesia (LID) affects up to 80% of patients after 5–10 years of treatment and has been associated with cognitive decline (9). In patients with Parkinson’s disease and mild cognitive impairment, the presence of levodopa-induced dyskinesia has been associated with a substantially higher risk of subsequent dementia (HR = 6.08; 95% CI 1.25–29.56) (10). These observations are consistent with studies linking dyskinesia to disruption of frontal–striatal networks involved in cognitive processing.

### 2.2 Levodopa-influenced psychosis

Psychosis, including hallucinations and delusions, affects up to 70% of patients after 20 years and is often triggered by dopaminergic therapy (11, 12). The onset of psychosis is a grim prognostic marker. It independently increases the risk of death by 71% (HR = 1.71; 95% CI 1.06–2.76) (13). Furthermore, psychosis is a direct driver of functional decline, associated with a 44% higher rate of falls and fractures (Incidence Rate Ratio = 1.44; 95% CI 1.39–1.49) (14) and a 49% increased risk of hospitalization (HR = 1.49; 95% CI 1.25–1.79) (15).

### 2.3 Levodopa and orthostatic hypotension (OH)

Orthostatic hypotension occurs in approximately 30–50% of patients with Parkinson’s disease and may be exacerbated by dopaminergic therapy (16). Orthostatic hypotension has been associated with increased fall risk (43–68%), higher mortality over 4 years (17), and a 285% increase in hospitalization days for falls, syncope, and related injuries (18).

### 2.4 Levodopa and weight loss

LID increases energy demands and triggers weight loss in PD (19, 20). As weight drops, levodopa dose per kilogram rises, worsening LID (20). Sustained underweight status doubles mortality risk (HR = 2.05; 95% CI 1.67–2.52), while severe weight loss more than triples it (HR = 3.36; 95% CI 1.60–7.08) (21).

### 2.5 Levodopa and impulse control disorders (ICDs) and dopamine dysregulation syndrome (DDS)

Levodopa significantly contributes to ICDs and is the most potent trigger of DDS, marked by compulsive medication overuse (22). These disorders have been associated with dysregulation of reward-related neural circuitry. ICDs impair cognitive function (Standard Mean Difference = −0.49; 95% CI -0.78 to −0.21) (23). DDS promotes compulsive overuse of levodopa, which fuels peak-dose dyskinesia, creating a vicious cycle of motor complications (24).

### 2.6 Levodopa and sleep disturbances

Levodopa may ease nocturnal akinesia (25), but chronic use disrupts sleep (26) and is linked to late-onset REM sleep behavior disorder (OR = 1.875; 95% CI 1.176–2.991) (27), which predicts aggressive PD progression, increasing cognitive decline risk by 80% (HR = 1.80) and motor progression by 37% (HR = 1.37) (28).

### 2.7 Metabolic pathway: homocysteine and methylation

Levodopa metabolism via catechol-O-methyltransferase (COMT) requires S-adenosylmethionine (SAMe) as a methyl donor. Chronic dopaminergic therapy therefore increases methyl group turnover, potentially reducing methylation reserve and elevating plasma homocysteine. Elevated homocysteine has been associated with endothelial dysfunction, vascular stiffness, peripheral neuropathy, and cognitive impairment in Parkinson’s disease. In parallel, reduced SAMe availability may impair global methylation processes relevant to neuronal maintenance and cellular resilience.

Recent reviews have emphasized that long-term levodopa exposure can increase homocysteine levels in a dose-dependent manner, particularly in the absence of adequate B-vitamin cofactor status (29, 30). This biochemical pathway may represent a potential systemic contributor within the proposed framework through which long-term dopaminergic therapy could interact with vascular and metabolic vulnerability to influence frailty trajectories (29, 30).

As seen in other fields such as cardiovascular disease, elevated homocysteine may also represent an epiphenomenon rather than a causal factor, and its role within this framework remains uncertain.

## 3 The multiplier effect: grounding “levodopa-accelerated frailty” in geriatric science

Within the proposed framework, the combined effects of these accelerants may act synergistically, producing what can be conceptualized as a multiplier effect on frailty risk (see Table 1). To justify this terminology, it is essential to ground it in the established principles of geriatric medicine. The most widely accepted operational definition is the Fried Frailty Phenotype, which defines frailty as a clinical syndrome characterized by the presence of three or more of the following five criteria: unintentional weight loss, self-reported exhaustion, weakness, slow walking speed, and low physical activity (31). The present hypothesis proposes that these seven accelerants may collectively map onto several components of the validated frailty phenotype.

**Table 1:** Conceptual distinction between disease-driven and treatment-associated contributors to frailty.

Domain	Disease-driven	Treatment-associated	Shared mechanisms
Motor	Progressive bradykinesia	Dyskinesia	Reduced mobility
Cognitive	Lewy pathology	LID-related network instability	Executive dysfunction
Autonomic	PD autonomic failure	OH exacerbation	Cerebral hypoperfusion
Metabolic	Sarcopenia	Hypermetabolism, homocysteine	Weight loss
Behavioral	Apathy	ICD/DDS	Reduced activity

### 3.1 Unintentional weight loss

Levodopa-induced dyskinesia (accelerant #1) creates a hypermetabolic state that leads to unintentional weight loss, directly fulfilling a key Fried frailty criterion (19, 20).

*Exhaustion*: (physical or systemic fatigue) may arise through the synergistic effects of sleep disturbance (accelerant #6), metabolic disruption (accelerant #7), including vascular stress and impaired methylation reserve, and the energy demands of persistent dyskinesia (accelerant #1) (9, 26, 27, 29).

### 3.2 Weakness, slow walking speed, and low physical activity

The motor and autonomic complications in PD reduce physical function. LID (accelerant #1) and orthostatic hypotension (accelerant #3) cause instability, unpredictable movements, and syncope risk, leading to slowness and reduced activity due to fear of falling (9, 16, 17). This is worsened by sarcopenia, which contributes to frailty in PD (7).

This constellation of deficits creates a state of heightened vulnerability through several synergistic mechanisms. The non-physiological, pulsatile stimulation from oral levodopa simultaneously dysregulates motor circuits (driving LID), reward circuits (driving ICDs/DDS), and perception circuits (driving psychosis), creating a Dopaminergic Overstimulation Triad (9, 11, 12, 22, 23). The path to dementia is accelerated by a Cognitive “Perfect Storm,” where chronic cerebral hypoperfusion from OH weakens the cognitive substrate, upon which the aberrant network activity of LID and the neurochemical chaos of psychosis impose a multiplicative risk (10, 13, 32). Finally, the Fall-Risk Cascade, amplified by the synergy of postural instability from OH, movement instability from LID, and perceptual instability from psychosis, creates a direct pathway to hospitalization, a known catalyst for catastrophic decline in PD (9, 14–18). Within the proposed framework, these interacting complications may contribute to a cascade of vulnerability that progressively reduces physiological reserve. Given how levodopa-related complications systematically worsen key elements of frailty, the term levodopa-accelerated frailty is proposed. This is not just a cluster of side effects, but a treatment-driven decline in physiological reserve. The clinical devastation of this state is underscored by findings that frailty itself has been associated with increased risk of dementia in Parkinson’s disease, increasing the odds of its development nearly threefold (OR = 2.91; 95% CI 1.54–5.99) (33).

## 4 Discussion: re-contextualizing clinical evidence and therapeutic strategy

This hypothesis fully aligns with the neutral disease-modification findings of the LEAP and ELLDOPA studies. The framework proposed here addresses a different, downstream mechanism: the *indirect* acceleration of frailty via the *cumulative burden of clinical complications*.

**Figure 1:**
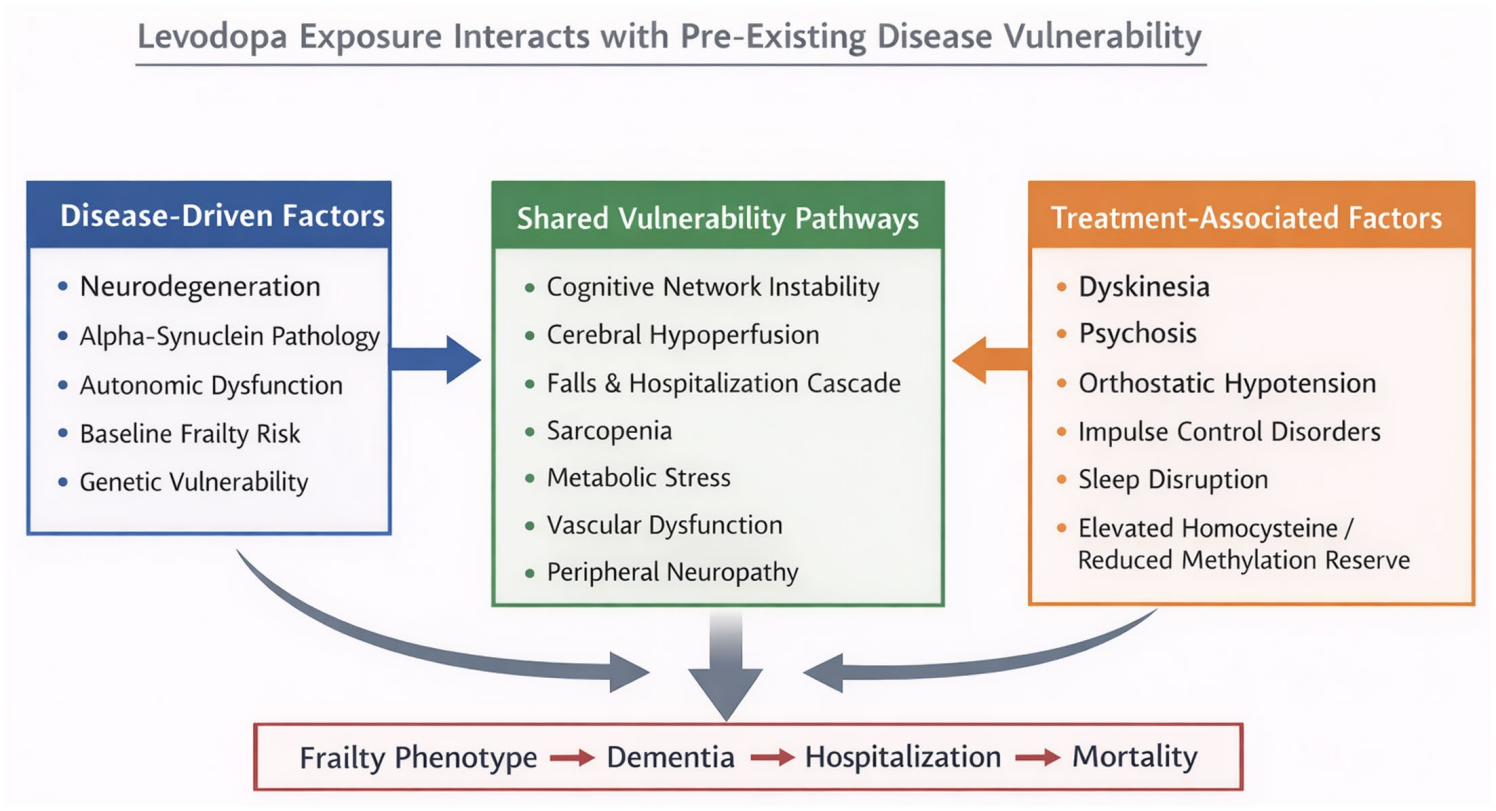
Conceptual model of levodopa exposure interacting with disease-related vulnerability pathways. Parkinson’s disease progression reflects the interaction between disease-driven factors (left), treatment-associated factors (right), and shared vulnerability pathways (center). Levodopa-associated complications and underlying neurodegenerative processes may converge on common mechanisms—including network instability, autonomic dysfunction, metabolic stress, and vascular impairment—that contribute to frailty development. Within the proposed framework, these interacting factors may collectively increase vulnerability to adverse outcomes, including dementia, hospitalization, and mortality. The model is hypothesis-generating and does not imply direct causality.

It is important to explicitly distinguish disease-driven processes from treatment-associated effects. Parkinson’s disease itself is a progressive multisystem disorder that independently leads to frailty. The present framework does not posit levodopa as a primary causal agent of frailty, but rather as a potential amplifier of vulnerability through interaction with pre-existing disease mechanisms. The conceptual distinction between intrinsic neurodegeneration, treatment-associated complications, and shared downstream pathways is illustrated in Figure 1 and Table 1.

Throughout this manuscript, the associations described between treatment-associated complications and adverse outcomes are interpreted as hypothesis-generating relationships rather than evidence of direct causality.

## 5 Distinguishing levodopa-accelerated frailty from advanced PD frailty

Frailty is common in advanced Parkinson’s disease and can arise directly from progressive neurodegeneration, autonomic dysfunction, and reduced mobility. The concept of *levodopa-accelerated frailty* does not imply that levodopa creates frailty *de novo*, but rather that cumulative dopaminergic exposure may amplify vulnerability through treatment-associated complications that are partially dose-dependent. In this framework, disease progression provides the underlying substrate for frailty, while long-term pulsatile dopaminergic stimulation may contribute additional systemic stressors, including dyskinesia, orthostatic hypotension, metabolic imbalance, and neuropsychiatric complications, that accelerate the transition from vulnerability to clinical frailty. The hypothesis therefore proposes an interaction between intrinsic disease progression and treatment exposure rather than attributing frailty solely to either mechanism.

The LEAP study’s finding that levodopa does not modify the underlying rate of neurodegeneration is crucial, as it supports the hypothesis that the observed long-term harm originates not from the drug’s effect on the primary pathology, but from these secondary complications (3).

It is essential to contextualize this framework alongside the key levodopa trials. The LEAP and ELLDOPA studies remain foundational evidence demonstrating the strong symptomatic benefits of levodopa while showing no evidence that the therapy accelerates underlying neurodegeneration. Our argument does not dispute these findings but highlights that these trials were not designed to assess late-stage, systemic complications, such as frailty, falls, or metabolic decline, that emerge after a decade of exposure. Therefore, while levodopa’s short-term safety is well established, its cumulative impact on long-term resilience remains an open question deserving prospective investigation.

As such, their neutral findings on disease modification cannot be used to rule out the cumulative, iatrogenic burden proposed here.

PD MED, the largest pragmatic trial of initial PD therapy, found levodopa produced a small but sustained PDQ-39 mobility benefit over 7 years compared with levodopa-sparing strategies (2). However, this came with a significantly higher incidence of dyskinesia in the levodopa-first group (2). Importantly, the study was open-label and designed primarily to assess symptomatic outcomes rather than long-term systemic consequences such as frailty trajectories.

The frailty hypothesis does not contradict the important findings of PD MED but rather seeks to place them within a multi-decade clinical trajectory, particularly for Young-Onset PD (YOPD) patients. It is posited that the PDQ-39, while an excellent measure of quality of life, may not fully capture the latent risk for accelerated cognitive decline, which may be associated with levodopa-induced dyskinesia and its link to progressive frontal network dysfunction (10). The trial’s median seven-year follow-up may be insufficient to observe the full clinical manifestation of this cognitive risk, which this hypothesis suggests becomes a dominant driver of “healthspan,” the years lived with functional independence, in the second and third decades of the disease.

## 6 Theoretical extrapolation: implications for young-onset Parkinson’s disease

The following discussion represents a theoretical extrapolation of the proposed framework, rather than a conclusion derived from longitudinal trial data. To illustrate the potential long-term implications of this hypothesis, consider a hypothetical YOPD patient facing decades of treatment. Under a traditional approach focused on maximal early symptom control, greater cumulative dopaminergic exposure has been associated with earlier emergence of dyskinesia and neuropsychiatric complications in Parkinson’s disease (9, 22). This model suggests that such a trajectory could translate to a higher risk of dementia and mortality in the second decade of the disease, consistent with the known prognostic impact of these complications. Conversely, a treatment strategy that limits cumulative dopaminergic exposure could hypothetically delay the onset of this proposed frailty cascade, potentially preserving healthspan and improving long-term outcomes, a possibility that warrants prospective investigation. These projections highlight the uncertainty surrounding cumulative treatment exposure and reinforce the need for long-duration randomized trials specifically designed to evaluate frailty trajectories and late-stage outcomes.

## 7 Addressing the duration vs. dose debate

It is critical to differentiate the *onset* of complications from their *severity*. Studies such as the Italian-Ghanaian cohort show that the *onset* of motor complications is strongly linked to *disease duration*, regardless of when levodopa is initiated (34). This paper’s hypothesis does not dispute this. However, observational studies have repeatedly reported that the severity of several levodopa-associated complications, particularly dyskinesia and metabolic effects, increases with cumulative or higher daily dopaminergic exposure (7, 20). This framework is focused on this *modifiable, dose-dependent* component of harm.

## 8 Re-evaluating dyskinesia: from ‘troublesome’ symptom to prognostic marker

A common clinical viewpoint regards levodopa-induced dyskinesia (LID) as an inevitable and often tolerable trade-off for avoiding debilitating ‘off’ periods. This perspective, however, may overlook its prognostic significance. As this hypothesis highlights, LID is a powerful predictor of cognitive decline, associated with a six-fold increased risk of dementia in patients with PD-MCI. This association suggests LID is not merely a ‘troublesome’ motor symptom but a clinical marker of aberrant frontal-striatal network activity that contributes to cognitive decline. This association raises the possibility that strategies limiting severe dyskinesia could have implications for long-term cognitive health, although this remains to be tested empirically.

## 9 Balancing iatrogenic risk with clinical necessity

This framework must be situated within clinical reality. Uncontrolled ‘off’ periods represent an immediate and profound danger, increasing fall risk and functional disability. Furthermore, the primary alternatives to levodopa, dopamine agonists and MAO-B inhibitors, are demonstrably inferior for motor control and carry their own significant burden of side effects, as seen in the PD MED trial. This hypothesis, therefore, does not advocate for a return to ‘levodopa phobia’. Rather, it calls for a re-evaluation of the therapeutic goal, shifting from maximal symptom suppression (which requires high, pulsatile dosing) to the lowest effective dose that maintains functional independence.

## 10 Clinical controversies and unresolved questions

If the hypothesis proposed here has merit, several important clinical questions arise that remain unresolved. These controversies highlight why long-term randomized trials evaluating cumulative dopaminergic exposure are needed.

### 10.1 Therapeutic goals

Should Parkinson’s disease therapy prioritize maximal short-term symptom suppression, or long-term preservation of physiological reserve and healthspan?

### 10.2 Cumulative dopaminergic exposure

Does minimizing cumulative dopaminergic exposure, potentially through intensive exercise and other non-pharmacological strategies (36), meaningfully reduce long-term complications, or does early aggressive symptom control ultimately yield better outcomes?

### 10.3 Lowest effective dose

Is the widely cited “lowest effective dose” principle clinically meaningful in long-term disease trajectories, and how should such a threshold be defined or measured?

### 10.4 Early detection of complications

Could earlier recognition of treatment-associated complications alter long-term frailty trajectories, or are these complications primarily markers of underlying disease progression?

### 10.5 Role of advanced therapies

Should device-based therapies be introduced earlier to reduce long-term pulsatile dopaminergic stimulation, or does current practice appropriately balance risk and benefit? (35). These unresolved questions illustrate the uncertainty surrounding cumulative dopaminergic exposure and underscore the need for adequately powered long-term randomized trials.

## 11 Toward an operational definition of levodopa-accelerated frailty

To move from conceptual framework to empirical testability, a preliminary operational definition is required. We propose that “levodopa-accelerated frailty” may be operationalized as the convergence of:

- A validated frailty measure exceeding a defined threshold (e.g., Fried phenotype ≥3 criteria or Frailty Index ≥0.25), and
- High cumulative levodopa exposure, quantified as lifetime cumulative levodopa equivalent daily dose (LEDD) or sustained high daily LEDD above a predefined threshold,
- In the absence of advanced disease stage alone fully accounting for frailty severity.

In research settings, this could be examined using longitudinal cohorts by modeling frailty trajectories as a function of cumulative LEDD while adjusting for disease duration, baseline motor severity, age, and comorbidities.

Such an operationalization does not assume causality but provides a measurable framework for testing whether cumulative dopaminergic exposure independently correlates with accelerated frailty progression.

## 12 Limitations and future directions

This article presents a conceptual framework rather than causal proof. The main limitation of this framework lies in distinguishing the effects of levodopa from the natural course of severe PD. Patients requiring higher doses may be biologically prone to faster decline, making levodopa a marker, not necessarily a cause, of deterioration.

The extent to which levodopa accelerates frailty is unclear. It may add only modestly to disease progression or significantly drive late-stage decline via its complications. Even small effects carry weight at the population level, while larger ones could radically reshape treatment strategies. Clarifying this impact is therefore both a scientific and ethical priority.

The hypothesis presented here also provides a potential framework for future randomized clinical trials examining long-term dopaminergic treatment strategies. While trials such as ELLDOPA, LEAP, and PD-MED have established the short-term symptomatic benefits of dopaminergic therapy, they were not designed to evaluate cumulative systemic outcomes such as frailty, hospitalization, or long-term functional independence. A long-duration randomized study comparing treatment strategies that differ in cumulative dopaminergic exposure could therefore clarify whether treatment-associated complications meaningfully influence frailty trajectories. Such trials would complement existing evidence while testing the hypothesis proposed in this manuscript.

Resolving this question demands prospective studies that stratify patients by baseline risk and track cumulative drug exposure alongside clinical outcomes. Current arguments rely on correlational data and require validation through such research.

The design of such trials raises important ethical considerations. Because levodopa is highly effective for symptomatic relief, long-term placebo-controlled studies may appear difficult to justify. However, levodopa has not been shown to alter the underlying neurodegenerative process, and its benefits are primarily symptomatic. From this perspective, a carefully designed randomized trial evaluating cumulative dopaminergic exposure, including strategies that delay or minimize exposure, could be ethically permissible if patients are closely monitored and rescue therapy is available when clinically necessary. Such designs have precedents in delayed-start and strategy trials in Parkinson’s disease. Nevertheless, the ethical balance between symptom control and the need to understand long-term treatment effects remains an important topic for discussion in the field.

## 13 Implications for future randomized clinical trials

Beyond observational cohort analyses, the hypothesis presented here ultimately requires testing through long-term randomized clinical trials, designed to evaluate the cumulative impact of dopaminergic exposure on frailty trajectories. Such studies would complement existing trials that establish the short-term symptomatic benefits of levodopa therapy, including ELLDOPA, the delayed-start LEAP trial, and the pragmatic PD-MED trial. A future randomized design could compare strategies emphasizing minimal effective dopaminergic exposure versus standard symptom-maximization approaches while prospectively measuring frailty indices, cognitive outcomes, hospitalization rates, and functional independence over extended follow-up. While such trials would be challenging to conduct, they represent the most rigorous approach to determining whether cumulative dopaminergic exposure contributes to accelerated frailty in Parkinson’s disease.

Future studies must prioritize long-term, holistic outcomes. Tools like the Fried Phenotype and Frailty Index should be used to quantify iatrogenic burden and frailty trajectories across diverse patient subgroups. Composite endpoints will be critical to identifying therapies that preserve not just life, but its quality.

## 14 Conclusion

Resolving the questions raised in this manuscript ultimately requires long-term, adequately powered randomized clinical trials designed to evaluate the cumulative effects of dopaminergic therapy on frailty trajectories and long-term functional outcomes in Parkinson’s disease. The hypothesis presented here provides a conceptual framework for why such trials are needed.

The levodopa paradox, that our most effective symptomatic therapy may also contribute to long-term vulnerability, invites a new, testable research paradigm. Viewing levodopa complications as a synergistic web of progression drivers shifts care from reactive symptom control to proactive, multi-decade strategy. Focusing on minimizing cumulative iatrogenic burden and preserving reserve through “levodopa-accelerated frailty” helps patients and clinicians navigate levodopa’s trade-offs more wisely (8, 33).

This critical question can be answered with existing resources. Large-scale datasets such as PD MED, PPMI, and other longitudinal cohorts already contain the necessary variables – cumulative levodopa dose, motor severity scores, weight, orthostatic vitals, and cognitive measures – to model the relationship between levodopa exposure and frailty trajectories while adjusting for disease severity. A dedicated secondary analysis of these datasets could clarify the effect size far more precisely than is currently possible and should be prioritized given its implications for millions of patients worldwide.

While this model remains a hypothesis, it provides a clear and testable roadmap for future research aimed at extending not just the lifespan, but the healthspan, of individuals living with Parkinson’s disease.

Ultimately, confirmation or refutation of this hypothesis will require prospective cohort analyses and, ideally, randomized clinical trials specifically designed to examine long-term frailty outcomes in relation to cumulative dopaminergic exposure.

## Data Availability

The original contributions presented in the study are included in the article/Supplementary material, further inquiries can be directed to the corresponding author.
