# Supplementary material for: Levodopa-accelerated frailty: a hypothesis for a cumulative iatrogenic burden in Parkinson’s disease
Source: Front Neurol. 2026 Apr 15;17:1798514. doi: 10.3389/fneur.2026.1798514 (PMC13126730; doi:10.3389/fneur.2026.1798514)
Supplement: Supplementary file 1 [file Table_1.docx]

**Table 1:** Risk Multipliers for Adverse Outcomes Associated With Iatrogenic Complications in Parkinson’s Disease

| **Complication** | **Adverse Outcome** | **Risk Multiplier (HR/OR/IRR)** | **95% Confidence Interval** | **Primary Source (Citation)** |
| --- | --- | --- | --- | --- |
| LID (in MCI) | Dementia | HR=6.08 | 1.25–29.56 | Lee JY, et al. 2019 [9] |
| Psychosis | Mortality | HR=1.71 | 1.06–2.76 | Stang C, et al. 2022 [12] |
| Psychosis | Falls/Fractures | IRR=1.44 | 1.39–1.49 | Forns J, et al. 2021 [13] |
| Psychosis | Hospitalization | HR=1.49 | 1.25–1.79 | Turcano P, et al. 2023 [14] |
| Sustained Underweight | Mortality | HR=2.05 | 1.67–2.52 | Yoon SY, et al. 2024 [20] |
| Excessive Weight Loss | Mortality | HR=3.36 | 1.60–7.08 | Yoon SY, et al. 2024 [20] |
| Levodopa Use (vs. no use) | Worsening RBD | OR=1.875 | 1.176–2.991 | Cao R, et al. 2022 [26] |

This table quantifies the increased risk for adverse outcomes associated with long-term progression of Parkinson’s disease. Each row presents the risk multiplier conferred by a specific complication induced or exacerbated by chronic levodopa therapy. The data are drawn from multiple sources.

**Abbreviations:**

LID, Levodopa-Induced Dyskinesia;

MCI, Mild Cognitive Impairment;

HR, Hazard Ratio;

OR, Odds Ratio;

IRR, Incidence Rate Ratio.
